# Characterization of the complete chloroplast genome sequence of *Deutzia glabrata* (Saxifragaceae)

**DOI:** 10.1080/23802359.2020.1715862

**Published:** 2020-01-21

**Authors:** Qian Yang, Cheng Xin

**Affiliations:** Key Laboratory of Resource Biology and Biotechnology in Western China (Ministry of Education), College of Life Sciences, Northwest University, Xi’an 710069, China

**Keywords:** *Deutzia glabrata*, chloroplast genome, phylogenetic analysis

## Abstract

*Deutzia glabrata* belongs to the Saxifragaceae, usually perennial herbs, shrubs. The cpDNA of *Deutzia glabrata* was 157,283 bp long with a large single-copy region (LSC) of 86,839 bp and a small single-copy region(SSC) of 18,748 bp separated by a pair of inverted repeat regions (IRs) of 25,848 bp. It contains 131 genes, including 85 protein-coding genes, 38 tRNA genes, 8 rRNA genes, of which 16 genes are duplicated in the IRs. The overall GC content is 37.6%. The phylogenetic tree indicates that *Deutzia* species formed a monophyletic lineage with high bootstrap value. This study has provided new genome information for the phylogenetic analysis of Saxifragaceae.

Saxifragaceae is a family of dicotyledonous plants and it is widely distributed in the world. *Deutzia glabrata* belongs to the Saxifragaceae and flowering in summer, white flowers, long flowering period, flowers are dense and elegant, suitable for planting on hillsides, lawns, roadsides, and can also be used as hedges. It is a good beautification and greening tree species in northern China. At present, the research on *Deutzia glabrata* is almost blank, and there is no analysis in molecular genetic evolution. In this study, we determined the complete chloroplast genome sequences of *Deutzia glabrata* to provide new genome information for the phylogenetic analysis of Saxifragaceae.

Total genomic DNA was isolated from a single individual of *Deutzia glabrata* sampled from Benxi (Liaoning, China; 40.39°N, 124.01°E). The voucher (2018XIN27) was deposited in the Evolutionary Botany Laboratory (EBL), Northwest University. Genomic DNA was isolated from the silica-dried leaves of a single individual with the improved CTAB method (Doyle 1987) and sequenced by using the Illumina HiSeq 2500 platform. Raw reads were trimmed by NGSQC Toolkit_v.2.3.3 (Patel and Jain 2012) and the clean reads were assembled by MITObim v1.8 (Hahn et al. 2013). The complete chloroplast genome was annotated by Geneious R8.0.2 (Biomatters Ltd., Auckland, New Zealand) with *Deutzia crassifolia* (MG524993) as the reference. The annotated genome has been deposited into GenBank with the accession number of MN872800.

The cpDNA of *Deutzia glabrata* is 157,283 bp in length with a large single-copy region (LSC) of 86,839 bp and a small single-copy region(SSC) of 18,748 bp separated by a pair of inverted repeat regions (IRs) of 25,848 bp. It contains 131 genes, including 85 protein-coding genes, 38 tRNA genes, 8 rRNA genes, of which 16 genes are duplicated in the IRs. The overall GC content is 37.6%, while the corresponding values in the LSC, SSC, and IR regions are 35.7, 31.3, and 43.0%, respectively. Phylogenetic relationships were presented using 23 published species. Their whole chloroplast genome sequences were aligned with the program MAFFT 7.308 (Katoh and Standley 2016) and adjusted manually. Maximum likelihood (ML) analyses were implemented in RAxML version 7.2.6 with 1000 bootstrap replicates (Stamatakis 2006). The phylogenetic tree indicates that *Deutzia* species formed a monophyletic lineage with high bootstrap value (Figure 1).

**Figure 1.**
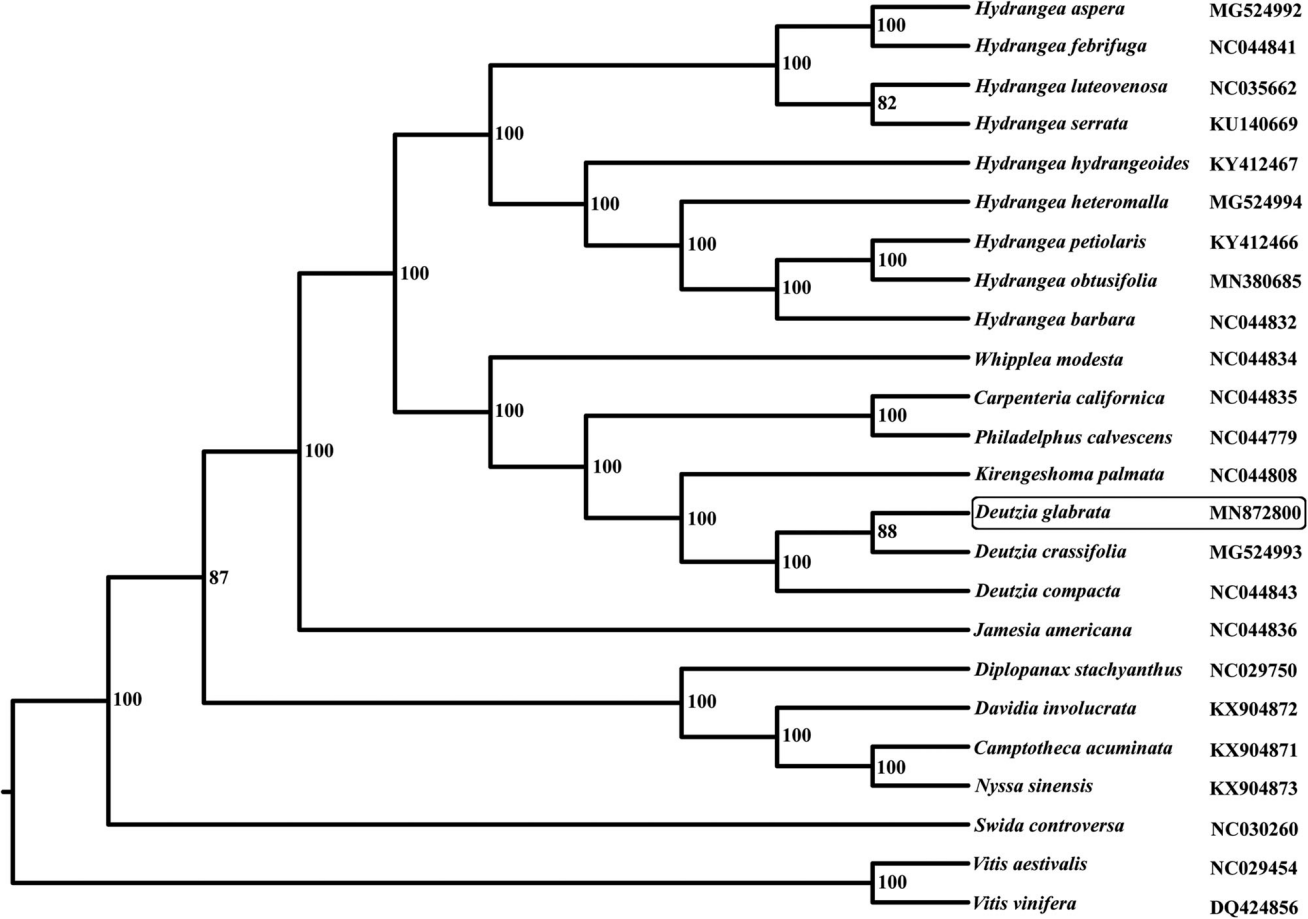
The phylogenetic tree based on complete chloroplast genome sequences.
